# Honey Bee Queens and Virus Infections

**DOI:** 10.3390/v12030322

**Published:** 2020-03-17

**Authors:** Esmaeil Amiri, Micheline K. Strand, David R. Tarpy, Olav Rueppell

**Affiliations:** 1 Department of Biology, University of North Carolina at Greensboro, Greensboro, NC 27402-6170, USA; o_ruppel@uncg.edu; 2 Department of Entomology & Plant Pathology, North Carolina State University, Raleigh, NC 27695-7613, USA; drtarpy@ncsu.edu; 3 Life Sciences Division, U.S. Army Research Office, CCDC-ARL, Research Triangle Park, NC 27709-2211, USA; micheline.k.strand.civ@mail.mil

**Keywords:** honey bee viruses, queen quality, virus transmission, worker attractiveness, IAPV, virus quantification, transgenerational effect, colony health, pathological symptom, virus monitoring

## Abstract

The honey bee queen is the central hub of a colony to produce eggs and release pheromones to maintain social cohesion. Among many environmental stresses, viruses are a major concern to compromise the queen’s health and reproductive vigor. Viruses have evolved numerous strategies to infect queens either via vertical transmission from the queens’ parents or horizontally through the worker and drones with which she is in contact during development, while mating, and in the reproductive period in the colony. Over 30 viruses have been discovered from honey bees but only few studies exist on the pathogenicity and direct impact of viruses on the queen’s phenotype. An apparent lack of virus symptoms and practical problems are partly to blame for the lack of studies, and we hope to stimulate new research and methodological approaches. To illustrate the problems, we describe a study on sublethal effects of Israeli Acute Paralysis Virus (IAPV) that led to inconclusive results. We conclude by discussing the most crucial methodological considerations and novel approaches for studying the interactions between honey bee viruses and their interactions with queen health.

## 1. Introduction

The high levels of honey bee colony losses call for research to identify all of the factors involved in the honey bee health crisis [1,2]. Among the many possible causes involved in colony weakness and death [3,4,5] poor “queen quality” is often reported by beekeepers as a principle contributing factor [2,6,7]. The queen is a critical member of the honey bee colony, and her primary roles are prolific egg production and the release of pheromones that maintain social cohesion [8,9,10]. In addition to the queen’s genetic make-up, queen quality―including health status and reproductive vigor―is a consequence of the nurse bees’ care during development of the queen larva, the health and fertility of the ~15 drones with which she mates, and the care she receives from worker bees in her colony after mating [11,12,13,14,15,16,17,18,19]. Simultaneously, queen health and reproductive capacity have direct impacts on the health, well-being, and vigor of the colony she is heading [8,20,21]. In many cases, failure or loss of the queen leads to colony mortality, especially outside of the reproductive season [3,6,22,23].

Although the queen is thought to be protected by several social immune mechanisms in the colony, she is exposed to different environmental stresses during different life stages that possibly have negative effects on her health and quality [24]. Initially, a queen can be influenced by the environment that her parents experienced as a result of a trans-generational stress response [25,26,27,28]. She can also be influenced by different environmental stressors during development, either through exposure to biotic or abiotic stressors in the colony environment or specifically via the quality or quantity of royal jelly she receives [29,30,31,32,33]. After maturation, a queen may face different stresses during her nuptial flights [33,34,35] and after the onset of oviposition inside her colony [36,37,38,39,40,41,42,43]. Environmental stresses can cause negative consequences for the developing queens such as substantial reduction in survival, and reduce queen emergence [29,31]. They may also compromise queens’ reproductive anatomy and physiology, egg laying, locomotor activity and behavior [32,33,40], and lower mating frequency [34]. At the molecular level environmental stresses can alter the activity of xenobiotics, oxidative stress and detoxification enzymes, negatively affect the immune competence of queens [41,42,43] and increase disease susceptibility [41,42]. The biotic stressor like viruses and Nosema can be transmitted venereally [35], infect the queens and possibly increase queen supersedure [38].

Among the different environmental stressors, viruses are considered to be a major concern across different stages of a queen’s life [24,44,45]. Viruses are obligate intracellular parasites that constantly evolve strategies to subvert their host cellular environment and co-opt host functionality. Here, we review the current literature on viral infections of queens followed by the description of one of our own studies of Israel Acute Paralysis Virus (IAPV) effects in queens. We discuss this study that led to inconclusive results on potential IAPV effects on queen attractiveness and trans-generational immunity to highlight some of the potential challenges with empirical research on the phenotypic effects of viruses on honey bee queens.

## 2. Viral Transmission Modes to Honey Bee Queens

Understanding transmission routes and directionality of viral spread is a crucial first step in determining the effect and epidemiology of a given pathogen, because transmission routes have direct effects on the prevalence and virulence of viruses [46,47,48]. Virulence is a term that has been used in insect pathology. In this text, we use virulence defined as “the disease producing power of an organism, the degree of pathogenicity within a group or species” [49]. Generally, horizontal transmission leads to the evolution of more virulent forms of the pathogen with negative impacts on host survival and fitness. In contrast, vertical transmission relies on host survival and reproduction. Therefore, it generally causes reduced virulence, allowing for long-term maintenance of viruses in host populations [47,48,50]. The relationship between transmission and virulence is more complicated when the pathogen has multiple transmission routes that can facilitate its spread [51]. Honey bee viruses can typically be transmitted through multiple means: vertically through eggs and stored sperm, horizontally via trophallaxis, glandular secretions, direct body contact, and vectors, or venereally through mating [45,47]. Accordingly, honey bee queens can be infected with viruses at the beginning of development from infected sperm or infected ovaries [52,53,54,55]. Simultaneous surveys of queens and their eggs indicate that many viruses detected from queens, such as Deformed Wing Virus (DWV), Sacbrood Virus (SBV), Black Queen Cell Virus (BQCV), and Kashmir Bee Virus (KBV) are also present in their eggs [56,57]. Other viruses, including Chronic Bee Paralysis Virus (CBPV), Acute Bee Paralysis Virus (ABPV), Israeli Acute Paralysis Virus (IAPV), and Lake Sinai Virus (LSV) have also been detected in honey bee eggs [54,55,58,59], which suggests that these viruses can also be vertically transmitted. In most cases, the prevalence of viruses in eggs can vary widely across colonies and populations. Although the majority of egg samples that have been assayed are infected only with one virus, most typically DWV, SBV, or BQCV, multiple virus infections are also common in honey bee eggs [54,55]. Any given fertilized virus-infected egg could potentially be reared into a queen and, consequently, carry and vertically transmit the virus infection for her entire lifespan. However less is known about the pathogenicity of viruses that are transmitted through eggs.

Several honey bee viruses, including DWV, BQCV, SBV, KBV, and ABPV, have been detected at the early larval stages in queens [56,57,60]. These viral infection(s) can originate either from queens’ parents (vertically transmitted; see above) or from nurse worker bees (horizontally transmitted) through infected food from glandular secretions [45]. Several viruses including IAPV, CBPV, DWV, and SBV have been detected from mandibular and hypopharyngeal glands of worker bees [58,59,61], which suggests that nurse bees can develop latent virus infection and transmit viruses into the royal jelly food that they provide to queens [57,59,60,62,63]. Consumption of virus particles in brood food by queen larvae (SBV, BQCV, KBV, IAPV) can cause infection and may kill the larvae during the larval or pupal stages if sufficiently large numbers of virus particles are ingested [60,64]. It should be noted that nurse bees are able to accumulate biological active RNAs including miRNAs, transposable elements, and non-coding RNA in the royal jelly that play a role in social immunity and are presumably active against pathogens such as viruses [62]. Also, the accumulation of virions (BQCV, DWV, and IAPV) in the wax of queen cells (and the possibility of subsequent virus transmission) should not be discounted during late larval or early pupal stages when a queen’s body is in contact with the wax [65]. Unlike in workers and drones, where horizontal transmission is greatly facilitated by the parasitic mite *Varroa destructor* that vectors many different viruses [66,67,68], queen larvae cannot acquire viruses from *Varroa* because they do not infest developing queen cells, except in exceedingly rare cases of colonies with very high *Varroa* mite infestation [69,70]. Developing queens with any disease symptoms are likely to be destroyed by worker bees, which selects for low virulence but could also be a direct reason why viruses are detected so sporadically in the different stages of queen development or in newly emerged queens [60].

Mating seems to be an important transmission route for viruses to infect queens. Multiple viruses, such as DWV, ABPV, BQCV, and SBV, have been detected from collected seminal fluid of apparently healthy drones, providing evidence that queens can be infected by mating or instrumental insemination [71,72]. Drones are favorable hosts for *Varroa* mites, which could elevate virus titers and the prevalence of viruses in drones. The viral load of drones directly affects queen health through venereal transmission of many viruses [45]. Among the viruses detected in queens, DWV has been investigated systematically to understand the venereal transmission to infect the queen and consequently vertical transmission from queen to produced eggs in honey bees [35,52]. DWV is a known cause of colony mortality in association with its vector, *Varroa destructor* [73], and it also serves as a convenient study model because it readily infects all developmental stages and castes [74]. DWV venereal transmission was demonstrated through instrumental insemination [52,53] and in free- flying mated queens [75,76]. High DWV titer detected from endophalli collected from captured drones from the drone congregation areas or from queens returning from their mating flights support that DWV venereal transmission can occur during natural mating [35,77]. As a result of venereal transmission, young mated queens are infected with more viruses than young unmated queens [35,44,78]. Older mated queens tend to be infected with yet a greater number of viruses, including DWV, SBV, BQCV, and AKI complex (ABPV, KBV, and IAPV), at higher infection titers [44,56,79]. In many cases, the same viruses that are detected in queen tissues can also be detected among workers of the same colony, suggesting that workers can infect the queen through trophallaxis or body contact [56,79]. Virus transmission through close bodily contact between infected workers and queens was shown directly for IAPV [80]. Queen pheromones attract retinue worker bees to surround the queen and interact with her by antennating, grooming, and feeding [81,82]. Since retinue workers are typically young (and therefore have little contact to the external hive environment), they may provide a physical and social barrier to protect the queen from disease and lower her exposure to infectious agents [62,83,84,85]. Nevertheless, queens are exposed, and older queens have had more interaction with worker bees throughout their longer life in the colony, accumulating viruses in an age- dependent manner. Whether immuno-senescence contributes to the higher virus titers of older queens is an open question.

## 3. Direct Health Impacts of Viruses on Queens

So far, over 30 viral pathogens have been reported to infect honey bees [86,87], some of which have only been identified and characterized recently [87]. In the absence of biotic (e.g., *Varroa* mite) and abiotic (e.g., exposure to pesticides, poor nutrition) stressors, most virus infections remain covert, without causing any clinical signs or symptoms either at the individual or the colony level [86,88]. However, once triggered, an overt virus infection can cause behavioral, physiological, and anatomical changes, including deformity, paralysis, or death [74,89]. With the development of molecular techniques, it becomes easy to detect and quantify viruses from entire queens or specific body parts, but the link between virus titers and pathological symptoms is not well understood in honey bee queens and in general. CBPV for example, causes two different forms of syndromes, independent of *Varroa*, but possibly related to other stressors [90]. In particular, pathological effects of viruses in queens are not well defined, because pathological signs are difficult to observe from a single individual that lives in the center of the colony and is presumably replaced quickly by her nestmates with a new queen when health-compromised. Therefore, direct health impacts of queens have only been studied for few viruses—typically in the laboratory or in older queens with advanced infections [44,70,91].

Consequently, little is known about how virus infections affect the behavior, physiology, reproduction, and longevity of queens. Numerous viruses (DWV, SBV, CBPV, ABPV, KBV, and IAPV) have been detected from heathy looking queens, demonstrating that infection can occur either by single or multiple viruses in a queen [75,76,78,92]. Viruses infect different parts of the queen’s body, including the head, thorax, ovaries, spermatheca, and fat body [56,59,79,93]. Although viruses can be detected from all body parts and tissues, some viruses have a considerable degree of tissue specificity. For example DWV seems to be concentrated in the reproductive tissues, the ovaries [52,93] and spermatheca [79] but was also directly observed in fat body cells [93]. IAPV also infects a variety of tissues and life history stages but is found at particularly high concentration in gut, ovaries, and spermatheca of infected queens [59].

Different studies indicate that highly virulent viruses, such as viruses in the AKI complex, are rarely detected in young unmated queens but occasionally found in older queens with low titers [44,60,76,79]. This pattern might arise because these virulent viruses kill the nurse worker bees and therefore are rarely transmitted to developing queens. Additionally, they rapidly kill any infected young queens. Older queens may be infected through physical contact that could lead to less virulent infection [80,91]. Alternatively, these viruses might be more commonly discovered in older queens because viral titers of covert infections that are initially below the detection limit may increase with age. In contrast, less virulent viruses (e.g., DWV, SBV, and BQCV) can readily be detected from unmated and young queens, and their prevalence and titers typically increase with age to considerable levels without killing or leading to the removal of the queens [35,44,79].

Several viruses, including DWV, SBV, and ABPV, have been detected from queen larvae or pupae [35,60], but the most noticeable pathological signs of virus infection during development are caused by BQCV, which is known as the most common cause of queen larval death [64,94]. BQCV infection arrests development at various stages, leading to a pale-yellow appearance and the presence of a sac-like skin. After death, the larva progressively blackens, giving the queen cell a darkened and “oily” appearance. In contrast, adult queens do not suffer from overt symptoms, even though they can harbor high titers of BQCV [56].

Specific clinical symptoms in queens have been studied for CBPV and DWV. Even though colonies can collapse with apparently healthy queens due to CBPV infection [90], CBPV injection, feeding, and topical infections of queens can cause trembling legs, spread and disjunct wings, and sometimes bloated abdomens full of hemolymph and dilated honey sacs [91]. Pathological DWV symptoms in queens seem to be restricted to colonies with a highly abundant *Varroa* population that causes high DWV viral titer. In these cases, queens can exhibit crippled wings [70] and degenerate ovaries, albeit without strict correlation between DWV titer and ovary degradation [44]. DWV infection was also found to be negatively correlated with stored sperm count [75], which may cause queen supersedure.

## 4. Case Study of IAPV Effects on Queen Attractiveness and Immune Priming

In an effort to expand our knowledge on sublethal effects of viral infections in honey bee queens, a study was conducted to test the hypotheses that virus infection reduces queen attractiveness and that offspring of virus-exposed queens better survive inoculation with the same virus due to inter- generational immune priming effects. Even though this study’s outcomes remained inconclusive, we describe it to highlight potential approaches and methodological innovation needed for overall progress in understanding the effects of viruses on their honey bee hosts.

The hypothesis of reduced queen attractiveness is based on findings that virus infection can change the cuticular hydrocarbon profile, leading to the recognition and removal of sick individuals [95,96]. Queen-worker interactions are modulated by the queen to protect herself from IAPV-infected workers [80]. However, it is not clear whether viruses can change worker behavior towards infected queens.

The second hypothesis of trans-generational survival benefits of a previous virus exposure is motivated by findings of such immune priming in honey bees against bacterial disease [25]. IAPV can cause significant changes in DNA methylation and transcriptional patterns within infected worker bees, potentially affecting protein levels and the functional response to infection [59,97]. It is plausible that inducible defense mechanisms against viruses exist in honey bees that could be used for immune priming offspring against IAPV. Immune priming against viruses has been reported from other insects [98] but no data exist in honey bees.

Thus, experimental queens were generated by standard queen rearing protocols from apparently healthy colonies [99]. After mating and the onset of reproduction, experimental queens were either inoculated with IAPV or sham treated. Several days post-treatment, the attractiveness of IAPV-exposed and control queens was compared by recording the behavioral choice of individual workers in a two-way olfactometer (see Supplemental File S1 for details). After the preference tests, IAPV-exposed and control queens were reintroduced into their colonies to produce worker offspring that was compared for survival of a topical IAPV inoculation under laboratory conditions. At the end of the study, the IAPV titers of all remaining queens were quantified by quantitative real-time PCR (see Supplemental File S1 for details).

**Figure 1:**
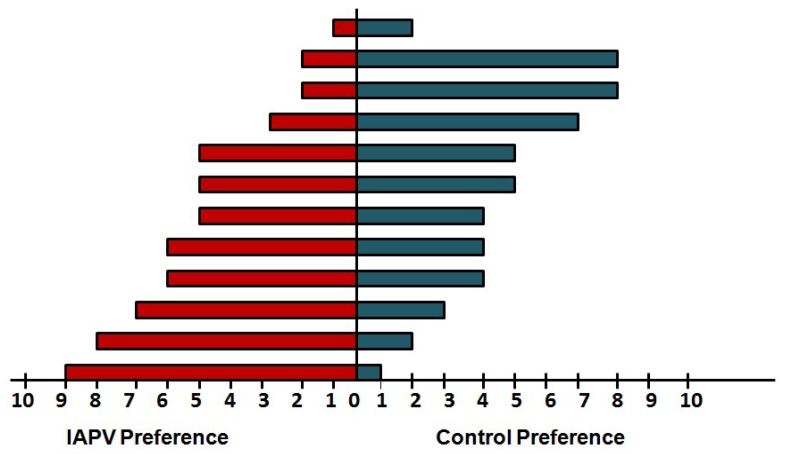
Worker preferences for Israeli Acute Paralysis Virus (IAPV)-exposed or control queens. For each queen pairing, ten independent workers were placed in a two-way olfactometer to choose between odors coming from an IAPV-inoculated (left) and sham-treated control queen (right) for ten minutes. Although workers chose the IAPV-inoculated queen significantly more than the control queen in one pairing (bottom bar), no overall preference according to experimental treatment was observed across all pairings.

Worker preference for control versus IAPV-exposed queens varied widely across experimental pairs (Figure 1). Contrary to our prediction, only one pairing indicated a significant worker preference and this preference was in favor of the IAPV-exposed queen. In the remaining trials, workers chose the IAPV-exposed queen more often than the control queen in five pairings, while the opposite was true in four pairings and the remaining two trials showed no bias.

**Figure 2:**
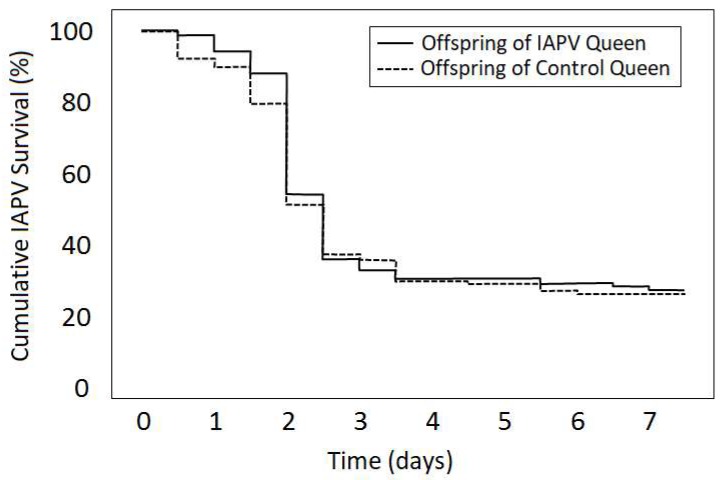
Survival of IAPV-inoculated offspring from IAPV-exposed and control queens. IAPV survival of workers produced by eight queens that were pre-exposed to IAPV was compared to workers from eight sham-treated control queens in laboratory cages. Despite significant variability among queens (data not shown), survival of offspring from inoculated queens was not significantly different from the survival of offspring from non-infected queens.

Overall, offspring of IAPV-exposed and sham-treated queens did not significantly differ in surviving an IAPV-inoculation (Kruskal–Wallis test: Χ^2^ = 0.7, N_primed_ = 134, N_unprimed_ = 128, p = 0.404; Figure 2). Individual queens were significantly different from each other (Log-rank test: Χ^2^ = 25.2, df = 15, *p* = 0.047), and offspring survival was more variable for sham-treated than for IAPV-exposed queens.

**Figure 3:**
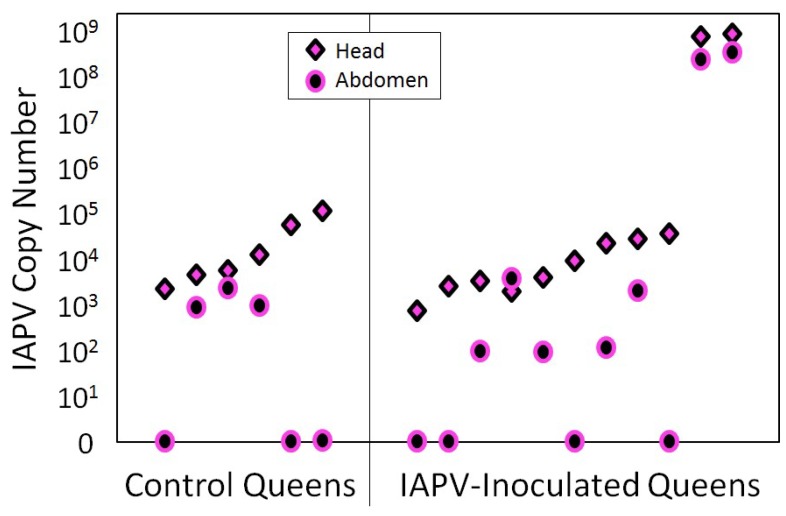
IAPV infection of the experimental queens after the conclusion of the experiment. IAPV was detected by RT-qPCR in all queens of the IAPV-inoculated and of the sham-treated control group, with no significant difference in IAPV quantities between treatment groups, although two of the IAPV-inoculated queens (right) had titers that were three orders of magnitude higher than the other queens.

At the end of the study, surviving queens were found with low levels of IAPV, regardless of treatment group. Only two queens from the IAPV-treated group were highly infected with IAPV at the end of the experiment (Figure 3).

## 5. Discussion

Queens emerge as a central concern for honey bee health and consequently have been receiving increased attention, particularly in relation to viral diseases [24,44,79]. With the help of next- generation sequencing, many new viruses have been documented in honey bees [87], but our knowledge of their distribution, infection routes, and pathogenicity in queens remains limited. Symptoms in queens differ from those in workers: frequently, viruses do not cause obvious symptoms in queens, perhaps to facilitate their own transmission. However, some overt infections have been documented that impair queen function, as discussed above [44]. Many current studies are focused on detection and transmission of viruses and significant progress in understanding viral epidemiology in honey bees is to be expected soon. More studies are especially needed to elucidate subtle virus effects and resolve the question of what triggers overt symptoms of honey bee viruses. Sublethal effects and symptoms are likely caste specific in honey bees, requiring specific studies on queens despite their difficulties.

Hoping to motivate methodological development and help others to plan the needed experiments, we described our case study because it illustrates many of these difficulties. The two most important problems with our above study are that our sham-treated control queens proved to contain IAPV at the end of the experiment and that only two of our IAPV-inoculated queens exhibited the expected high IAPV titers. Multiple explanations exist for both outcomes, even though all queens were grafted from the same, apparently healthy colony, IAPV may have been present below the detection level and increased titers may have been triggered by the experimental stress or other environmental factors. Alternatively, queens may have become infected during the pre-experimental period in their hives, which may have occurred during their natural mating [35,77] or a result of them sharing the experimental apiary with the IAPV-inoculated queens. Separated apiaries for infected and control queens might have reduced the infection risk for the control queens but could have introduced other potentially confounding differences between the experimental groups. The lack of expected high IAPV titers in the inoculated queens might indicate that our inoculation method was ineffective in this particular instance, even though preliminary experiments had been performed to ensure that our methodology would be effective. Alternatively, the majority of surviving queens may have successfully cleared the IAPV infection to low levels by the end of the experiment. Dosage and the temporal dynamics of the immune response are of clear concern for sublethal virus studies. A key method to enable these kinds of studies and to collect more conclusive data in the future is a technique to monitor virus titers in living individuals throughout an experiment. Fecal and egg sampling has been suggested [53,54,56,59] but in the case of feces, defecation is hard to induce at regular intervals and we envision an approach that is more akin to virus monitoring in vertebrates, although at a much smaller scale. Very small hemolymph samples should suffice for RT-qPCR, which is possible to perform even on single cells [100]. In addition, such a technique would be very useful to pre-screen all individuals in a study, not only for the virus under investigation, but also for other diseases that might otherwise obscure treatment effects.

Studies focusing on honey bee queens under normal conditions are necessarily limited in sample size because one queen necessitates the maintenance of an entire colony [10,84]. In our study, this led to the repeated use of several control queens. This form of pseudo-replication prevented an overall statistical analysis, a problem that can be prevented by careful planning of required resources and raising about 50% surplus queens to replace queens that die before the experiment can be completed. Low sample sizes in the preference tests of individual queen pairs also limited their statistical power but behavioral studies are very time consuming without automation of the assays. Likewise, the test of immune priming was plagued by low sample size; studies of mortality dynamics require much higher sample sizes and replicates distributed across many replicates because of the intrinsic stochasticity of mortality, particularly in laboratory cage studies [101,102]. Finally, the sampling schedule for collecting offspring may also have prevented us from finding an effect because immune priming can be transient [103] and offspring should have been tested at multiple time points after queen inoculation with IAPV.

In conclusion, the interactions between viruses and their honey bee queen hosts have practical importance for maintaining pollinator health because the queens play an important role in vertical transmission of many health-relevant viruses. More research is needed to document the distribution of viruses and their dynamics across space and time. Continued discovery of novel viruses or virus strains can be anticipated, necessitating continued monitoring efforts. This is particularly true to honey bee queen breeding operations that widely distribute their bees to their customers. Alternative to virus monitoring of queen breeders, local, small-scale queen breeding efforts could mitigate the risk of human-assisted virus spread over long distances. Beyond the practical importance of more virus research in honey bee queens, honey bees present unique opportunities of academic interest to study the relations between host physiology, virus transmission and replication, and virus pathogenicity. Specifically, the interplay between caste, potentially differential virulence evolution, and social immune mechanisms should be of great general interest. However, we believe that many experimental difficulties that complicate investigations into the interactions between viruses and honey bee queens need to be addressed before conclusive studies can improve our current understanding of queen–virus relationships and honey bee virology in general.

## Supplementary Materials

The following are available online at https://www.mdpi.com/1999-4915/12/3/322/s1, File S1: Case study of IAPV effects on queen attractiveness and immune priming: Experimental details.
