# Supplementary material for: Honey Bee Queens and Virus Infections
Source: Viruses. 2020 Mar 17;12(3):322. doi: 10.3390/v12030322 (PMC7150968; doi:10.3390/v12030322)
Supplement: Supplementary file 1 [file viruses-12-00322-s001.pdf]

Supplementary

Case study of IAPV effects on queen attractiveness and immune priming: Experimental details

Twenty first-instar larvae were grafted from a single source colony into queen cups and placed into a queenless rearing colony following established protocols [1]. Both colonies were apparently healthy and quantitative real-time PCR (RT-qPCR) testing for the presence of IAPV (see details below) were negative. Upon emergence, each queen was introduced into a mating hive with ~300-400 worker bees so that they could openly mate. After mating and the onset of oviposition, 12 queens were inoculated by topical application of approximately 10^8 IAPV particles in 2 μL as described by [2]. The other 8 queens were sham treated with distilled water. The attraction trials of IAPV-exposed and control queens were performed 2-12 days post treatment in a two-way olfactometer. A total of 12 queen pairs were assayed but four of the control queens were used twice, paired with different IAPV-exposed queens. Ten workers per queen pair were tested in separate trials (except for one trial with 9 and another with 3 workers). No overall statistical test was performed because of the lack of independence of control queens, but worker preference within individual pairings was analyzed by separate sign tests.

The two-way olfactometer consisted of five sectioned chambers of PVC was developed (Figure S1) to test the prediction that IAPV-exposed queens were less attractive than uninfected control queens. An aquarium pump with a two-way splitter connected to two tubes provided a slow and even air flow from both sides into the central chamber. Queens were placed on the opposing ends of the olfactometer in the outer chambers, and after an acclimation period of about five minutes, a single worker per trial was introduced into the central chamber. The orientation response of this workers was observed for 10 minutes under red-light to avoid visual orientation of the test subjects. The workers could cross from the middle chamber through a one-way wire-mesh funnel into either “selection” chamber, which was defined as the worker’s preference. Workers were 2 to 4-days old and collected from a colony in the UNCG apiary that had no relation to the tested queens. The placement of control and IAPV-inoculated queens were switched between the outer chambers after each trial to reduce any directional bias. Queens were fed a small amount of honey every 30 minutes during the experiment. Before each set of tested queens, the apparatus was disassembled and cleaned with bleach, 70% ethanol, rinsed with water, and dried. Significance of worker choice behavior was tested by calculating exact random probabilities of each observed distribution.

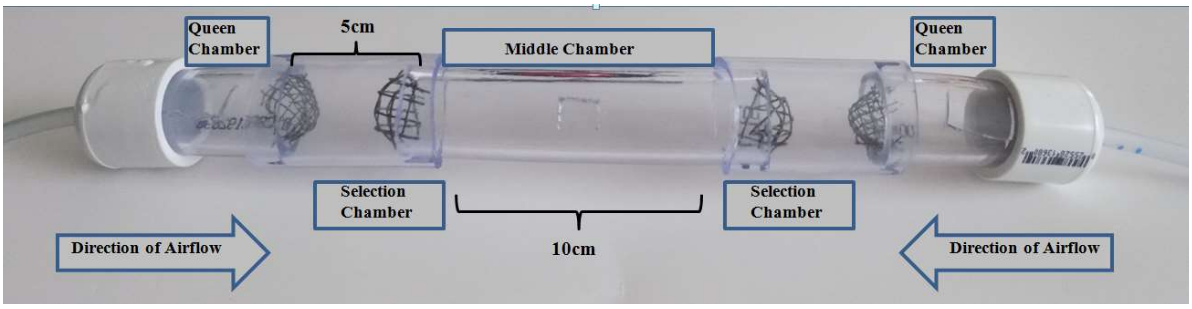

Figure S1. Design of the two-way olfactometer used in the worker attraction assay. One control and one IAPV-exposed queen were inserted at opposite queen chambers and, after an acclimation period, individual workers were introduced into the middle chamber to observe their orientation towards either queen. When a worker entered the selection chamber, the trial was terminated. No preference was recorded if the worker had not entered either selection chamber after ten minutes.

After the preference tests, IAPV-exposed and control queens were re-introduced into their respective hives, and after one month two frames of capped brood were collected and placed inside colony-specific

emergence cages within an incubator set at brood-nest conditions. Emerging adults were allowed to mature for four days before workers (n = 8-23 per queen) were collected from each of eight IAPV-exposed and eight control colonies and transferred into separate Plexiglass™ cages with ad libitum queen candy and water. All workers were inoculated with IAPV using topical application of approximately 10^8 IAPV particles in 2 µL as described above. The treated workers were returned to their cages and kept at 34 °C and ~60% RH for one week. Mortality was surveyed twice per day, and survival was compared between treatment groups by a generalized Kruskal-Wallis test across all queens. Additionally, all queens were compared individually, although this analysis may have limited validity because each queen's offspring was only surveyed in one or two replicate cages [3].

For eleven IAPV-exposed and eight control queens that survived until the end of the study, IAPV titers in head and abdomen were quantified with qRT-PCR as described previously [2] with slight modifications: total RNA was extracted with Trizol™, cDNA was synthesized with the SensiFAST™ cDNA Synthesis Kit, and IAPV titers were quantified by comparison to a standard dilution series. Each RT-qPCR was performed in duplicate, using the SensiFAST SYBR Green™ kit on a StepOnePlus™ cycler according to the manufacturer's recommendations. IAPV was assayed with forward 5′-CCATGCCTGGCGATTCAC-3′ and reverse 5′-CTGAATAATACTGTGCGTATC-3′ primers [4] and *A. mellifera* actin was used as positive control with the following primers: 5′ TTGTATGCCAACACTGTCCTTT 3′ and 5′TGGCGCGATGATCTTAATTT 3′ [5].

References

1. Büchler, R.; Andonov, S.; Bienefeld, K.; Costa, C.; Hatjina, F.; Kezic, N.; Kryger, P.; Spivak, M.; Uzunov, A.; Wilde, J. Standard methods for rearing and selection of *Apis mellifera* queens. *J. Apic. Res.* **2013**, *52*, 1-30, doi:10.3896/IBRA.1.52.1.07.
2. Amiri, E.; Seddon, G.; Zuluaga Smith, W.; Strand, M.K.; Tarpy, D.R.; Rueppell, O. Israeli acute paralysis virus: Honey bee queen–worker interaction and potential virus transmission pathways. *Insects* **2019**, *10*, 9, doi:10.3390/insects10010009.
3. Williams, G.R.; Alaux, C.; Costa, C.; Csáki, T.; Doublet, V.; Eisenhardt, D.; Fries, I.; Kuhn, R.; McMahon, D.P.; Medrzycki, P., et al. Standard methods for maintaining adult *Apis mellifera* in cages under *in vitro* laboratory conditions. *J. Apic. Res.* **2013**, *52*, doi:10.3896/ibra.1.52.1.04.
4. de Miranda, J.R.; Cordoni, G.; Budge, G. The Acute bee paralysis virus–Kashmir bee virus–Israeli acute paralysis virus complex. *J. Invertebr. Pathol.* **2010**, *103*, S30-S47, doi:1016/j.jip.2009.06.014.
5. Simone, M.; Evans, J.D.; Spivak, M. Resin collection and social immunity in honey bees. *Evolution* **2009**, *63*, 3016-3022, doi:10.1111/j.1558-5646.2009.00772.x.
